# Cardiotoxicity from bruton tyrosine kinase inhibitors (BTKi)—an analysis of an administrative health claims database

**DOI:** 10.1186/s40959-024-00237-x

**Published:** 2024-06-01

**Authors:** Srilakshmi Vallabhaneni, Srinath Adusumalli, Jingyi Wu, Peter W. Groeneveld, James Gerson, Rupal P. O’Quinn

**Affiliations:** 1 Cardiovascular Division, Department of Medicine, Dell Medical School, The University of Texas, Austin, TX USA; 2 CVS Health, Woonsocket, RI USA; 3 Division of General Internal Medicine, Department of Medicine, Perelman School of Medicine, University of Pennsylvania, Philadelphia, PA USA; 4 Corporal Michael J. Crescenz VA Medical Center, Philadelphia, PA USA; 5 Department of Oncology, University of Vermont, Burlington, VT USA; 6 Cardiovascular Division at Penn Presbyterian Medical Center, Department of Medicine, Perelman School of Medicine of the University of Pennsylvania, Philadelphia, PA USA; 7 University of PennsylvaniaPenn Presbyterian Medical Center Heart & Vascular Pavilion, 4th Floor 51 N. 39th Street, Philadelphia, PA 19104 215-662-9000 USA

**Keywords:** BTKi, Cardiotoxicity, Hypertension, Atrial fibrillation, Cardio-oncology, Arrhythmia

## Abstract

**Background:**

First generation Bruton tyrosine kinase inhibitors (BTKi) such as ibrutinib have been associated with cardiovascular toxicities. Newer generation BTKi (e.g.,acalabrutinib and zanabrutinib) have been associated with lower incidence of cardiotoxicity in clinical trials.

**Objective:**

Given paucity in real-world data on the overall cardiac risk factor profile, especially with the newer BTKi, our study evaluated the incidence of cardiotoxicity with various BTKi among a large, commercially insured population of patients.

**Methods:**

We performed a retrospective cohort analysis of all adults with a diagnosis of B-cell malignancy undergoing treatment with BTKi acalabrutinib and ibrutinib between January 2018 and June 2020 using Optum’s de-identified Clinformatics® Data Mart Database. We then identified patients who had pre-existing cardiac disease one year prior to starting BTKi. New incidence of atrial fibrillation/flutter, hypertension, bleeding, ventricular tachycardia/fibrillation and sudden cardiac death from the time of index presciption were compared with standard Chi Square or Student t-test where appropriate. Multivariate logistic regression models were also estimated to evaluate for confounding.

**Results:**

A total of 1691 patients were included in the final analysis. 1595 (94%, median age 75 (19–90) years, 61% male gender) patients received ibrutinib, and 96 (6%, median age 73.5 (32–90) years, 62.5% male gender) patients received acalabrutinib. The median duration of drug exposure of ibrutinib was 238 (2-1084) days vs. 150 (30–870) days for acalabrutinib. There was lower new incidence of atrial fibrillation/flutter (4.6%-vs-17%, *p* = 0.013), hypertension (6.3%-vs-25%, p = NS), sudden cardiac arrest/death (0% vs. 1.5%, p = NS) in the acalabrutinib group compared to ibrutinib, of which only the lower incidence of atrial fibrillation/flutter was statistically significant. This was despite the finding of a higher prevalence of atrial fibrillation/flutter at baseline in patients receiving acalabrutinib.

**Conclusions:**

There was lower incidence of new atrial fibrillation/flutter with acalabrutinib when compared to ibrutinib in a real-world cohort of patients.

**Supplementary Information:**

The online version contains supplementary material available at 10.1186/s40959-024-00237-x.

## Background

Chronic lymphocytic leukemia (CLL) is the most common adult leukemia and is characterized by accumulation of malignant mature B cells in the bone marrow, peripheral blood, lymph node and spleen [1]. Bruton’s tyrosine kinase (BTK) plays a crucial role in the survival and proliferation of leukemic cells in many B-cell malignancies, including CLL, small lymphocytic leukemia (SLL), diffuse large B-cell lymphoma, Waldenstrom’s macroglobulinemia, mantle cell lymphoma, marginal zone lymphoma, as well as chronic graft versus host disease [2–5]. The development of Bruton tyrosine kinase inhibitors (BTKi) has been a significant advancement in the treatment of CLL and related B-cell malignancies. BTKi have revolutionized treatment for B-cell malignancies due to higher efficacy in patients with high-risk features as well as better tolerability in elderly patients when compared to conventional chemotherapy [6]. However, BTKi have been shown to increase incidence of cardiac and vascular side-effects in clinical trials [7]. Ibrutinib was the first BTKi to be granted accelerated approval by US Food and Drug Administration (FDA) in 2013 after a landmark trial by Byrd et al. [8, 9]. Ibrutinib has proven to be highly efficacious in B-cell malignancies. However, atrial fibrillation (AF) emerged as an important treatment related side-effect that warranted closer inspection at its cardiotoxicity risk profile [7]. There are also reported incidence of hypertension, ventricular arrhythmias, including ventricular tachycardia, ventricular fibrillation and sudden cardiac death that can occur as soon as 65 days from initiation of ibrutinib [10, 11], with a median onset of 7.6 months for atrial fibrillation with BTKi [12]. This led to the development of acalabrutinib and zanubrutinib which are selective BTKi that demonstrated superior progression-free survival in a phase III clinical trial for patients with previously untreated or relapsed or refractory CLL [13, 14]. The phase III clinical trial also showed a lower incidence of AF/atrial flutter, hypertension, and cardiac events [15, 16] in acalabrutinib. A more recent paper by Brown JR et al. revealed that in patients with relapsed or refractory CLL/SLL, not only was progression-free survival significantly longer in patients that received zanubrutinib vs. ibrutinib, but that fewer cardiac adverse events were also seen in the former [17].

However, the overall side-effect profile of the selective BTKi is less clear given the lack of real-world data. In the present study, we sought to identify the incidence of cardiotoxicity among patients treated with a BTKi among a large, commercially insured population of patients.

## Methods

### Study cohort

Data for this study were obtained from Optum’s Clinformatics® Data Mart (CDM), a database of administrative health claims for members of large commercial and Medicare Advantage health plans. This database consists of claims data from inpatient facilities, outpatient facilities, providers, laboratories, and pharmacies consisting of a cohort of > 15 million patients annually.

**Fig. 1:**
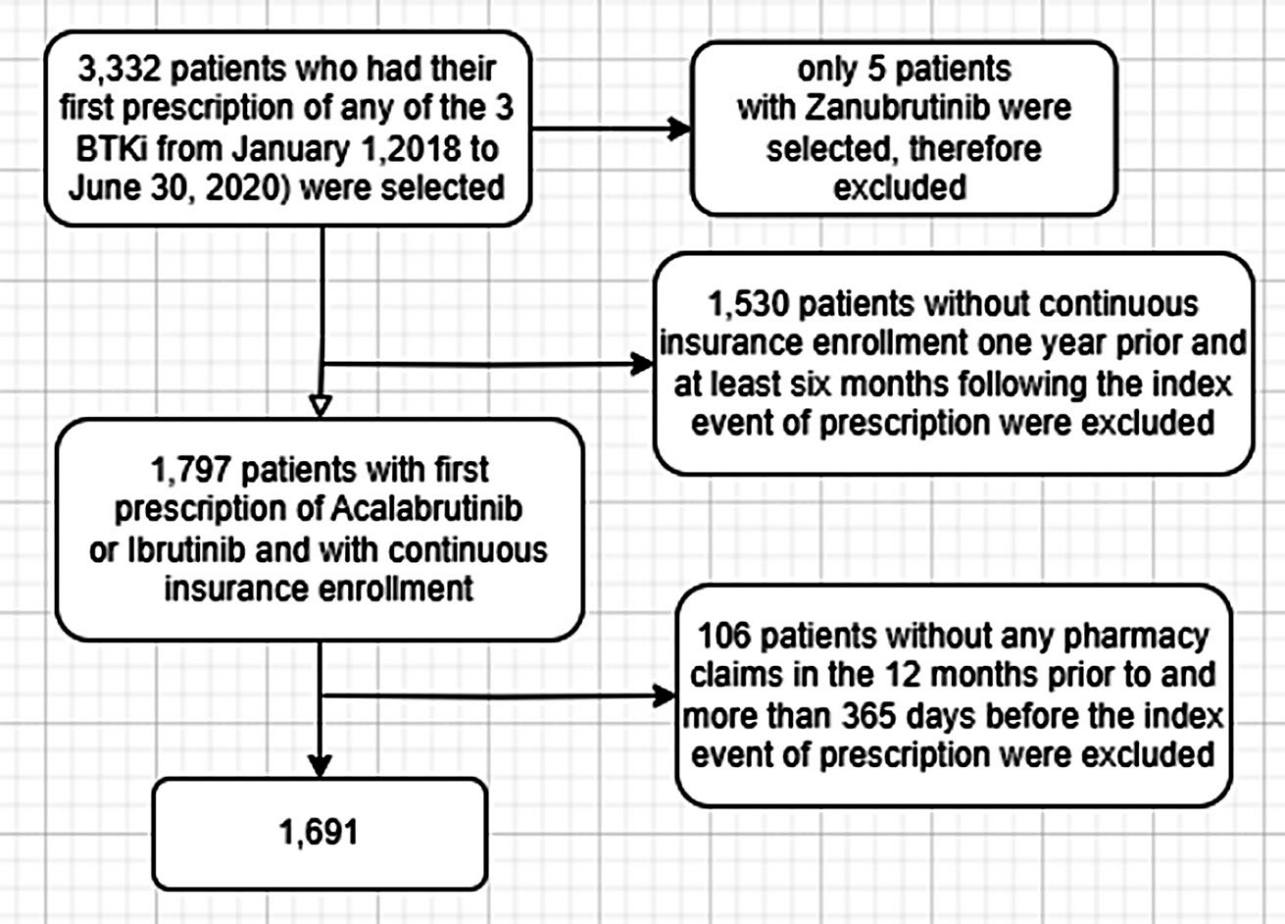
Study cohort algorithm

We identified all adult (age > 18 years) patients with a diagnosis of CLL, SLL, mantle cell lymphoma, Waldenstrom’s macroglobulinemia, marginal zone lymphoma, or “other” who were on a BTKi using the International Statistical Classification of Diseases, Ninth and Tenth Revision codes used to identify these diseases Supplemental Table 1). Patients who filled the first prescription for ibrutinib, acalabrutinib, or zanubrutinib in the database were identified from January 2018 until June 2020 to reduce selection bias and ensure that patients were on the BTKi for a similar duration. We then identified patients who had pre-existing cardiac disease including hypertension, atrial fibrillation/flutter, ventricular tachycardia/fibrillation, sudden cardiac arrest, as well as bleeding one year prior to starting BTKi using International Classification of Diseases, Ninth Revision, Clinical Modification (ICD-9-CM) and Tenth Revision, Clinical Modification (ICD-10-CM) (Supplemental Table 2). We excluded patients from the study if they did not have continuous insurance enrollment one year prior and at least six months following the index event of prescription. Patients without any pharmacy claims for 12 months prior to index event of prescription and patients without any pharmacy claims at least 365 days prior to the index event of prescription were also excluded. Figure 1 outlines the cohort study algorithm.

### Predictor variables

We collected baseline variables of age, gender, race, comorbidities including hypertension, hyperlipidemia, heart failure, history of myocardial infarction, obesity (Supplemental Table 3). Most patients were in the Midwest, South and Southeast. Demographic and socioeconomic data, including median household income, were available through zip code–linked enrollment data from the US Census Bureau. Race and ethnicity were determined in this database through a combination of public records, self-report, and proprietary ethnicity code tables.

### Outcome variables

The primary variable was incidence of new cardiotoxicity after starting BTKi including atrial fibrillation/flutter, hypertension, ventricular tachycardia/fibrillation, sudden cardiac arrest and bleeding in those that did not carry a prior diagnosis to better highlight that these toxicities occurred in patients that did not have a predilection for them.

### Statistical analyses

We compared patients who received their first acalabrutinib and ibrutinib during January 2018 to June 2020. For each group, we presented frequency and percentage for the summary statistics. Categorical variables were compared using Chi-square testing. Continuous variables were compared using Student t test. We estimated multivariate logistic regression models with the first diagnosis of atrial fibrillation/flutter, hypertension, bleeding, and ventricular arrhythmias as dependent variables for each model, and the independent variables is the medication type the patient was first prescribed and the covariates including age, gender, and comorbidities. Statistical analyses were performed using SAS, version 9.4 (SAS Institute Inc).

## Results

A total of 1691 patients were identified after applying the exclusion criteria. Of these, 1595 (94%) patients received ibrutinib, and 96 (6%) patients received acalabrutinib. Zanubrutinib was excluded when only 5 patients returned. Baseline characteristics are outlined in Table 1. Median age was noted to be 73.5 years ± 10.2 years (range 32–90 years) for acalabrutinib and 75 years ± 10.3 years (range 19–90 years) for ibrutinib group. Gender and baseline incidence of cardiac comorbidities was similar between both groups.

There was no statistical difference in the prevalence of hypertension (83%-vs-81%), bleeding (25%-vs-27%, p = NS), sudden cardiac arrest/death (1% vs. 0.8%, p = NS), ventricular tachycardia/fibrillation (4% vs. 4%, p = NS) prior to starting BTKi acalabrutinib vs. ibrutinib. However, there was higher prevalence of atrial fibrillation/flutter at baseline in patients receiving acalabrutinib compared to ibrutinib (31%-vs-22%, *p* = 0.030) (Table 2). After starting targeted therapy, 12/96 (13%) in the acalabrutinib arm and 409/1595 (26%) in the ibrutinib arm developed cardiotoxicities, as further detailed in Table 3. A patient may have developed more than one toxicity. The median duration of drug exposure was 150 (30–870) days in the acalabrutinib group, and 238 (2-1084) days in the ibrutinib group. The incidence of atrial fibrillation/flutter (4.6%-vs-17%, *p* = 0.013; odds ratio (OR) 4.4, 95% CI, 1.36–14.22, *p* = 0.013) was statistically higher in patients receiving ibrutinib compared to acalabrutinib. The incidence of specific toxicities such as hypertension (6.3%-vs-25%, p = NS) and sudden cardiac arrest/death (0%-vs-1.5%, p = NS) were lower in the acalabrutinib group compared to ibrutinib; however, these did not reach statistical significance. The incidence of ventricular tachycardia/fibrillation (2%-vs-2.5%, p = NS) and bleeding (10%-vs-13%, p = NS) was similar between the two groups. Of the 213 patients with atrial fibrillation/flutter while on ibrutinib, only 4 were switched to acalabrutinib.

**Table 1:** Baseline characteristics of patients receiving Ibrutinib and Acalabrutinib

	Acalabrutinib (*n* = 96)	Ibrutinib (*n* = 1595)	*p*-value
	Frequency (%)	Frequency (%)	
**Age group**			
18–54	3 (3)	74 (5)	0.754
55–64	16 (17)	216 (13.5)	
65–74	31 (32)	506 (32)	
≥75	46 (48)	799 (50)	
**Gender**			
Female	36 (37.5)	618 (39)	0.807
Male	60 (62.5)	977 (61)	
**Race**			
Asian	1 (1)	29 (2)	0.856
Black	7 (7)	131 (8)	
Hispanic	6 (6)	137 (9)	
White	67 (70)	1082 (68)	
Unknown/missing	15 (16)	216 (13.5)	
**Education level**			
Less than 12th grade	0 (0)	6 (0.4)	0.108
High school diploma	15 (16)	394 (25)	
Less than bachelor degree	46 (48)	764 (48)	
Bachelor degree plus	24 (25)	260 (16)	
Unknown/missing	11 (11.5)	171 (11)	
**Household income range**			
<$40K	18 (19)	317 (20)	0.618
$40K-$49K	8 (8)	94 (6)	
$50K-$59K	4 (4)	136 (8.5)	
$60K-$74K	11 (11)	161 (10)	
$75K-$99K	15 (16)	226 (14)	
$100K+	26 (27)	374 (23.5)	
Unknown/missing	14 (15)	287 (18)	
**Occupation type**			
Manager/owner/professional	4 (4)	88 (6)	0.874
White collar/health/civil service/military	5 (5)	86 (5)	
Blue collar	2 (2)	52 (3)	
Homemaker/retired	11 (11.5)	146 (9)	
Unknown/missing	74 (77)	1223 (77)	
**Federal poverty status**			
Above 400% FPL	82 (85)	1308 (82)	0.396
Below 400% FPL	0 (0)	0 (0)	
Unknown/Missing	14 (15)	287 (18)	
Comorbidities			
Obesity	28 (29)	384 (24)	0.259
HTN	80 (83)	1288 (83)	0.532
Dyslipidemia	70 (73)	1174 (74)	0.881
Systolic heart failure (heart failure with reduced ejection fraction)	13 (13.5)	111 (7)	0.016
History of myocardial infarction	11 (11.5)	150 (9)	0.505
Peripheral artery disease	32 (33)	471 (30)	0.428
Cerebrovascular disease	19 (20)	284 (18)	0.622
Chronic kidney disease	30 (31)	442 (28)	0.452

**Table 2:** Prevalence of cardiovascular events prior to initiation of BTKi

	Acalabrutinib (*n* = 96)	Ibrutinib (*n* = 1595)	*p*-value
	Frequency (%)	Frequency (%)	
Atrial fibrillation/atrial flutter	30 (31)	347 (22)	0.030
Hypertension	80 (83)	1288 (81)	0.532
Bleeding	24 (25)	417 (27)	0.804
Sudden cardiac arrest/death	1 (1)	12 (0.8)	0.753
Ventricular tachycardia/fibrillation	4 (4)	60 (4)	0.840

**Table 3:** Incidence of cardiovascular events after initiation of BTKi

	Acalabrutinib (*n* = 12)	Ibrutinib (*n* = 409)	*p*-value
	Frequency (%)	Frequency (%)	
Atrial fibrillation/atrial flutter	3 (4.6)	213 (17)	*0.013*
Hypertension	1 (6.3)	77 (25)	0.121
Bleeding	7 (10)	156 (13)	0.392
Sudden cardiac arrest/death	0 (0)	23 (1.5)	0.972
Ventricular tachycardia/fibrillation	2 (2)	38 (2.5)	0.856

**Fig. 2:**
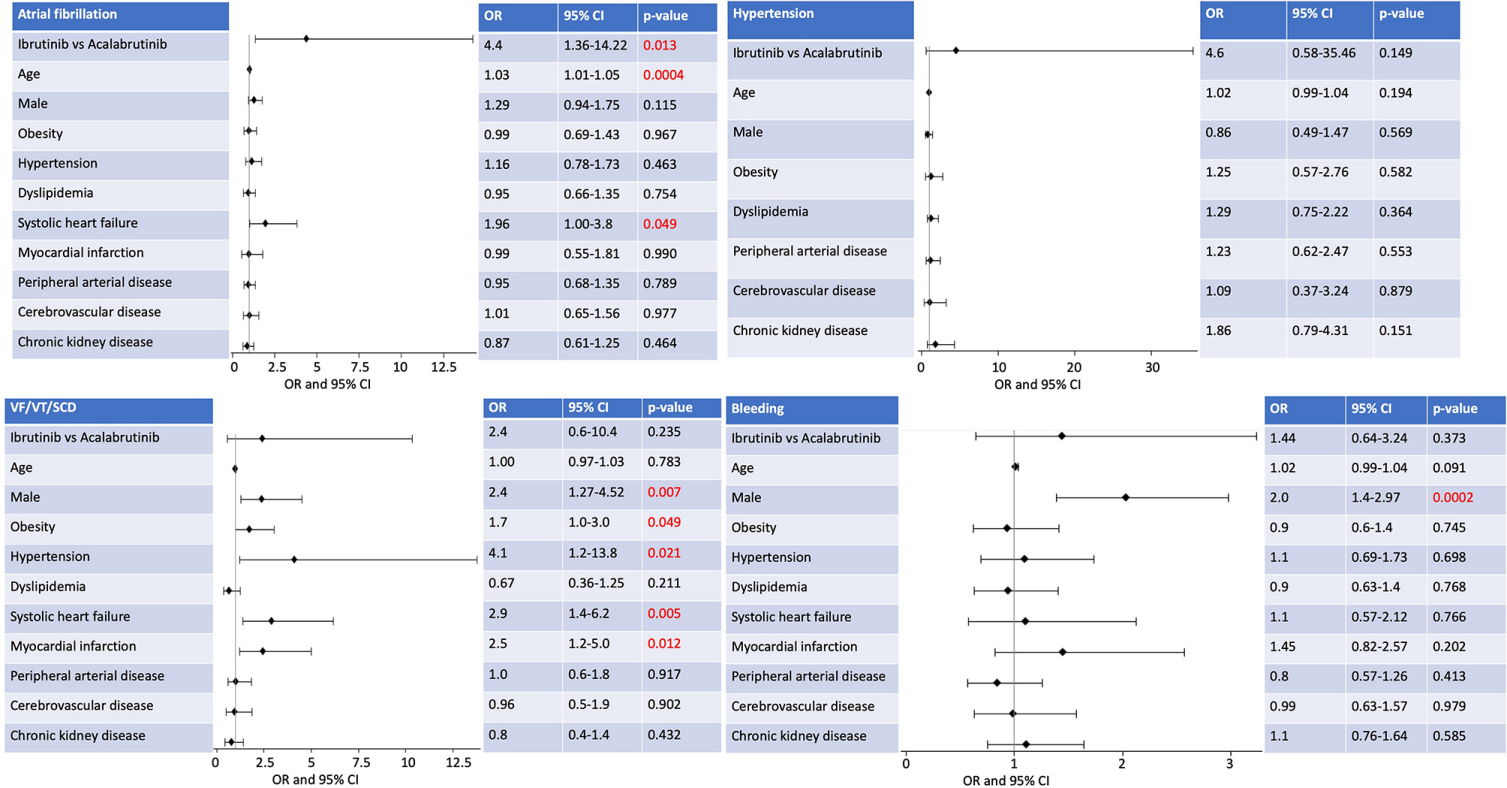
Primary endpoints of cardiotoxicity with BTKi in a multivariate analysis. After starting targeted therapy with BTKi, incidence of atrial fibrillation/flutter was higher in patients receiving ibrutinib compared to acalabrutinib [OR 4.4 (95% CI, 1.36–14.22, *p* = 0.013)]. Older patients and those with heart failure with reduced ejection fraction were more likely to be diagnosed with atrial fibrillation/flutter. Men had higher incidence of bleeding while on either BTKi compared to women. Male gender, obesity, hypertension, systolic heart failure and history of myocardial infarction increased the risk of ventricular tachycardia/fibrillation and sudden cardiac death. BTKi–bruton tyrosine kinase inhibitors, CI–confidence interval, OR–odds ratio, SCD–sudden cardiac arrest/death, VF–ventricular fibrillation, VT–Ventricular tachycardia

Multivariate analysis (Fig. 2) revealed that older patients [OR 1.03 (95% CI 1.01–1.05, *p* = 0.0004)] and those with heart failure with reduced ejection fraction [OR 1.96 (1.00–3.8, *p* = 0.049)] were more likely to be diagnosed with atrial fibrillation/flutter. Men had a higher incidence of bleeding while on either BTKi compared to women [OR 2.0 (95% CI, 1.4–2.97, *p* = 0.0002)]. Male gender, obesity, hypertension, systolic heart failure and history of myocardial infarction increased the risk of ventricular tachycardia/fibrillation and sudden cardiac arrest/death.

## Discussion

Ibrutinib is the first BTKi in its class, and its toxicity profile has been well characterized [18]. The cardiovascular toxicities include hypertension, supraventricular and ventricular arrhythmias, increased risk of bleeding and central nervous system hemorrhage [15, 16]. A study by Salem et al. also found that ibrutinib was also associated with a higher risk of heart failure, conduction disorders, CNS ischemic events in addition to these, when compared to all the other drugs in the Vigibase (International pharmacovigilance database) [16]. Another population-based cohort study by Abdel-Qadir et al. looking at Ontario patients found that ibrutinib was associated with a higher risk of AF, bleeding, and heart failure, but not acute myocardial infarction nor stroke [19].

In a retrospective cohort study of patients with CLL, development of AF was the most common cause of discontinuation of ibrutinib [20]. In a meta-analysis of four randomized clinical trials of patients with CLL, SLL, and mantle cell lymphoma treated with ibrutinib vs. non-ibrutinib therapy, the pooled incidence rate of AF in the ibrutinib group was 3.3 per 100 years compared to 0.84 per 100 person years in the non-ibrutinib group [21]. Avalon JC et al. also found that pre-existing cardiovascular disease was associated with a significantly higher rates of atrial fibrillation/arrhythmias as well as mortality in those receiving treatment with ibrutinib [22].

Recently, BTKi with higher selectivity for BTK have been developed and it has been postulated that more selectivity may lead to fewer off-target cardiovascular side effects [23, 24]. The phase III clinical trial comparing the safety and efficacy of ibrutinib to acalabrutinib showed lower incidence of AF/atrial flutter in the acalabrutinib treated patients compared to ibrutinib (9.4%-vs-16%, *P* = 0.02), hypertension (9.4%-vs-23.2%) with a total incidence of cardiac events being 24.1%-vs-30% [25]. Kaplan-Meier analyses of cumulative incidence showed hazard ratios of 0.52 (95% CI, 0.32 to 0.86) and 0.34 (95% CI, 0.21 to 0.54) favoring acalabrutinib for AF or atrial flutter and hypertension, respectively [25]. A pooled analysis of safety data from clinical trials with acalabrutinib in B-cell malignancies also showed lower incidence of hypertension and atrial fibrillation at 8% and 4% respectively [26]. However, trial data often do not reflect the complex co-morbidities of real-world patients. A paper by Bhat et al. found that ventricular arrhythmias and ventricular ectopy was 8-fold higher in an almost 300 patient cohort treated with acalabrutinib at Ohio State although the severity/grading of these events is unknown [27].

To our knowledge, this study is the first real-world study done comparing cardiovascular toxicities among various BTKi. In our study, we found lower incidence of atrial fibrillation/flutter with selective BTKi, acalabrutinib. There was lower incidence of hypertension and sudden cardiac death/arrest, although this did not meet statistical significance. Notably, the incidence of sudden cardiac arrest/death with acalabrutinib was noted to be 0%. It is possible that a small sample size in the acalabrutinib group played a role in the lack of statistical significance. One important point to note is that the mean age in our study was older in the 70s, versus median of 66 years in the phase III clinical trial comparing the two agents [24]. This may also be contributing to the increased prevalence of hypertension in our study population. Patients with significant cardiovascular disease were also excluded in that trial while > 20% of patients in this study had pre-existing AF. Acalabrutinib was approved in November 2019 for CLL and SLL based on two landmark clinical trials based on efficacy and progression free survival [13, 14], and ibrutinib’s approval nearly five prior years prior may explain the lower number of individuals in the acalabrutinib group since the Clinformatics® database currently only includes data up to June 2020 at the time of our analysis. Acalabrutinib was granted accelerated approval by the FDA for mantle cell lymphoma in October 2017, but this is a rare disease.

The prevalence of atrial fibrillation/flutter prior to initiation of BTKi was higher in the acalabrutinib group compared to ibrutinib (31% vs. 22%, *p* = 0.030, outlined in Table 2). One hypothesis is that oncologists may be more likely to use acalabrutinib in those that are felt to be at especially high risk of cardiotoxicity, creating selection bias. Despite the bias, there was a lower incidence of new atrial fibrillation after starting acalabrutinib compared to ibrutinib (4.6%-vs-17%, *p* = 0.013). The incidence is similar to that reported in clinical trial data of 4–9%. Atrial fibrillation with BTKi is thought by some to occur because of reduction in PI3K-AKT pathway signaling. This may be due to a direct off-target effect due to cross talk between BTK and PI3K-AKT pathways resulting in inhibition of the latter pathway [28, 29]. Xiao et al. suggest that atrial fibrillation may be caused by inhibition of C-terminal Src kinase based on a mouse study [30]. Acalabrutinib’s enhanced specificity for BTK and/or less inhibition of C-terminal Src kinase may cause lower off-target effects, explaining the lower incidence of atrial fibrillation in our cohort of patients.

Interestingly, only 4/213 (2%) of the patients with atrial fibrillation/flutter while on ibrutinib were switched to acalabrutinib. This low number is most likely due to the availability of alternative classes of therapies that carry no risk of atrial arrhythmias that can be used in lieu of BTKi. It may also be due to the slow adoption of a new treatment as seen with new therapies in general.

Given that multivariate analysis revealed that patients with heart failure with reduced ejection fraction were more likely to be diagnosed with atrial fibrillation, it may be worth considering a more selective BTKi especially in this population.

## Limitations

Our study has several limitations. First, the use of an administrative database limits in-depth details of the circumstances around individual patient care decisions. Second, the smaller sample size with acalabrutinib compared to ibrutinib is likely due to FDA approval for acalabrutinib occurring more recently in November 2019. It is possible that another analysis of the cohort available from June 2020 and beyond may yield a different result since acalabrutinib may be more widely used with time, especially since the National Comprehensive Cancer Network updated their Clinical Practice Guidelines in Oncology to include acalabrutinib as a Category 1 preferred treatment option (nccn.org). The median duration of drug exposure was also higher in the ibrutinib group, and thus this may have had an impact on the incidence of cardiovascular events, although we did attempt to keep drug exposure rates similar by assessing only new incidence of cardiovascular events within six months of BTKi initiation. However, the incidence of cardiovascular toxicities may increase with time, and thus may not be fully captured in our study. Another limitation is that CLL/SLL and the other B-cell malignancies primarily affect the elderly, many of whom are > 65 years of age and who are likely covered by some form of government-insured program such as Medicare. The incidence of atrial fibrillation and cardiovascular disease increases with age, and by excluding Medicare patients, this may exclude many patients at greater risk for cardiovascular side effects. The Optum database cohort thus may miss a large swath of the population within the United States that is receiving treatment with BTKi, although our cohort includes patients who are covered by Medicare Advantage. A large, prospective multi-center study is especially needed to evaluate the true incidence of cardiotoxicity with selective BTKi. Third, due to the small number of patients receiving zanabrutinib, this group was excluded from the study analysis. This may be especially relevant since phase III data suggests zanubrutinib has fewer off-target effects and less cardiotoxicity than ibrutinib [31, 32], similar to acalabrutinib. A future direction includes evaluating all three BTKi, as well as non-covalent BTKi that are in development such as pirtobrutinib [33]. Fourth, we evaluated incidence of only hypertension, atrial fibrillation/flutter, ventricular arrhythmias, sudden cardiac arrest/death, and bleeding due to lack of resources to assess other toxicities. However, there are other cardiotoxicities that have been associated with BTKi, such as conduction disorders and central nervous system hemorrhage specifically. Future studies are needed to evaluate the incidence of these toxicities with the various BTKi. Lastly, it is important to note that since we were only able to evaluate new incidence of cardiovascular toxicities, our data does not capture the full clinical picture since patients may have experienced worsening of their baseline cardiovascular co-morbidities, such as uncontrolled hypertension or recurrent arrhythmia, resulting in higher medication doses or changes.

## Conclusions

We found that there is lower incidence of new atrial fibrillation/flutter with acalabrutinib when compared to ibrutinib within six months of drug exposure. There was lower incidence of other cardiotoxicities (hypertension, sudden cardiac arrest/death), although findings did not reach statistical significance. More studies with a larger number of patients on more selective BTKi, such as acalabrutinb and zanubrutinib as well as any new agents, are needed to further delineate its role in the incidence of cardiotoxicity compared to the other BTKi. Larger prospective multi-centered trials are needed to confirm these findings and expand upon them.

### Electronic supplementary material

Below is the link to the electronic supplementary material.

Supplementary Material 1

## Data Availability

Proprietary data obtained and used from Optum’s Clinformatics® Data Mart (CDM). Data under contract with strict prohibitions against data sharing outside of our organization.

## Abbreviations

BTKi: Bruton tyrosine kinase inhibitors
CLL: Chronic lymphocytic leukemia
SLL: Small lymphocytic leukemia
AF: atrial fibrillation
HFrEF: heart failure with reduced ejection fraction
OR: odds ratio
