# Supplementary material for: Cardiotoxicity from bruton tyrosine kinase inhibitors (BTKi)—an analysis of an administrative health claims database
Source: Cardiooncology. 2024 Jun 1;10:33. doi: 10.1186/s40959-024-00237-x (PMC11143603; doi:10.1186/s40959-024-00237-x)
Supplement: Supplementary file 1 — Supplementary Material 1 [file 40959_2024_237_MOESM1_ESM.docx]

**Supplementary Information**

**Supplemental Table 1:**

*International Statistical Classification of Diseases, Ninth and Tenth Revision* codes used to identify chronic lymphocytic lymphoma (CLL)

| CLL | ICD-10-CM: C91.10, 91.11, 91.12, 95.11, 95.12 |
| --- | --- |
| SLL | ICD-10-CM: C83.00 |
| MCL | ICD-10-CM: C83.17, 83.10, 83.12, 83.13 |
| WM | ICD-10-CM: C88.0 |
| MZL | ICD-10-CM: C88.4 |

**Supplemental Table 2*:***

*International Statistical Classification of Diseases, Ninth and Tenth Revision* codes used to identify cardiovascular toxicities associated with BTKi

|  | **Diagnosis** | **ICD-9-CM** | **ICD-10-CM** |
| --- | --- | --- | --- |
| Hypertension | Hypertension  Malignant hypertension  Benign essential hypertension  Unspecified essential hypertension  Hypertensive heart disease  Malignant hypertensive heart disease without heart failure  Malignant hypertensive heart disease with heart failure  Benign hypertensive heart disease  Benign hypertensive heart disease without heart failure  Benign hypertensive heart disease with heart failure  Unspecified hypertensive heart disease  Unspecified hypertensive heart disease without heart failure  Unspecified hypertensive heart disease with heart failure | 401  401.0  401.1  401.9  402.0  402.00  402.01  402.1  402.10  402.11  402.9  402.90  402.91 | ICD-10-CM: I10.X, I11.X-I13.X, I15.X |
| Atrial fibrillation/Atrial flutter | Arrhythmia, NOS  Atrial fibrillation  Atrial flutter | 427.0  427.31  427.32 | I10, I15, I15.8, I15.9 |
| Sudden cardiac arrest | Cardiac arrest | 427.5 | I46.2, I46.8, I46.9 |
|  | Sudden death, cause unknown | 798 | - |
|  | Instantaneous death | 798.1 | - |
|  | Death occurring in less than 24 hours from onset of symptoms, not otherwise explained | 798.2 | - |
| Ventricular arrhythmia | (Paroxysmal) ventricular tachycardia | 427.1 | I47.2 |
|  | Ventricular fibrillation and flutter | 427.4 | I49.0 |
|  | Ventricular fibrillation | 427.41 | I49.01 |
|  | Ventricular flutter | 427.42 | I49.02 |
| Bleeding | Intracranial | 430, 431, 432.0, 432.1, 432.9 |  |
|  | Upper gastrointestinal | 531.0, 531.2, 531.4, 531.6, 532.0, 532.2, 532.4, 532.6, 533.0, 533.2, 533.4, 533.6, 534.0, 534.2, 534.4, 534.6, 578.0, 578.1, 578.9 | K92.0, K92.1, I85.0, I98.20, I98.3, K22.10, K22.12, K22.14, K22.16, K25.0, K25.2, K25.4, K25.6, K26.0, K26.2, K26.4, K26.6, K27.0, K27.2, K27.4, K27.6, K28.0, K28.2, K28.4, K28.6, K29.0, K63.80, K31.80. |
|  | Lower gastrointestinal | 569.3 | K55.20, K62.5, K92.2 |
|  | Other Bleeding | 287.8, 287.9, 596.7, 784.8, 599.7, 627.1, 459.0, 719.1, 786.3 | N02.0, N02.1, N02.2, N02.3, N02.4, N02.5, N02.6, N02.7, N02.8, N02.9, K66.1, N93.8, N93.9, N95.0, R04.1, R04.2, R04.8, R04.9, R31.0, R31.1, R31.8, R58, D68.3, H35.6, H43.1, H45.0, M25.0 |

**Supplemental Table 3:**

*International Statistical Classification of Diseases, Ninth and Tenth Revision* codes used to identify patient comorbidities

| Obesity | ICD-10-CM: E66.X |
| --- | --- |
| HTN | ICD-10-CM: I10.X, I11.X-I13.X, I15.X |
| Dyslipidemia | ICD-10-CM: E78.0X, E78.2, E78.4, E78.5 |
| Systolic heart failure (heart failure with reduced ejection fraction) | I50.2X, I50.4X |
| History of myocardial infarction | ICD-10-CM: I21.X, I22.X, I23.X, I25.2 |
| Peripheral Artery Disease | ICD-10-CM: I70.x, I71.x, I73.1, I73.8, I73.9, I77.1, I79.0, I79.2, K55.1, K55.8, K55.9, Z95.8, Z95.9 |
| Cerebrovascular Disease | ICD-10-CM: I63.X, I65.X, I66.X, I67.X, I69.3X |
| Chronic kidney disease | ICD-10-CM: I12.0, I13.1, N18.x, NI9.x, N25.0, Z49.0, Z94.0, Z 99.2 |

**NDC codes for BTK inhibitors:**

Acalabrutinib (3): 0310-0512-28, 0310-0512-60, 0310-0512-95

Zanubrutinib (2): 72579-011-01, 72579-011-02

Ibrutinib (9):  57962-014-28, 57962-070-28, 57962-140-09, 57962-140-12, 57962-280-28, 57962-420-28, 57962-420-71, 57962-560-28, 57962-560-71
